# P-Cadherin (CDH3) is overexpressed in colorectal tumors and has potential as a serum marker for colorectal cancer monitoring

**DOI:** 10.18632/oncoscience.370

**Published:** 2017-10-21

**Authors:** H.M.C. Shantha Kumara, Geoffrey A. Bellini, Otavia L. Caballero, Sonali A.C. Herath, Tao Su, Aqeel Ahmed, Linda Njoh, Vesna Cekic, Richard L. Whelan

**Affiliations:** ^1^ Division of Colon and Rectal Surgery, Department of Surgery, Mount Sinai Roosevelt Hospital Center, New York, NY, USA; ^2^ Ludwig Institute for Cancer Research Ltd, New York Branch of Human Cancer Immunology at Memorial Sloan-Kettering, New York, NY, USA; ^3^ Herbert Irving Comprehensive Cancer Center, Columbia University, New York, NY, USA; ^4^ Icahn School of Medicine at Mount Sinai, New York, NY, USA; ^5^ Current address: Orygen Biotecnologia S.A., São Paulo, Brazil

**Keywords:** CDH3, colorectal cancer, immunotherapy target

## Abstract

**Introduction:**

Placental-Cadherin (CDH3) is a cell adhesion molecule vital to cellular localization and tissue integrity. It is highly expressed in the placenta (PLC)and is overexpressed by many types of cancer. P-cadherin levels in colorectal cancer (CRC) remains poorly characterized. This study's purpose was to determine P-cadherin expression in CRC and normal tissues and to assess plasma CDH3 levels in order to determine the relationship, if any, between cancer stage, P-cadherin expression and plasma CDH3 levels.

**Methods:**

An IRB approved plasma, tumor, and prospective data bank was utilized. CRC patients for whom tumor and normal colon tissue samples were available were enrolled. Tumor samples were OCT embedded and stored at -80C°. Total purified RNA was isolated from tissue samples and cDNA synthesized. CDH3 expression was analyzed by quantitative PCR (QPCR) using the SYBR Green platform. Tumor expression levels were determined and compared to levels in normal colonic tissue and PLC. Expression in 22 different normal tissues was also assessed by RT-PCR. Plasma CDH3 levels were determined via ELISA in patients for whom preoperative blood samples were available. Results: A total of 77 paired CRC and normal colon specimens (36 M/ 41 F, age 67.3±14.5) were assessed (82% colon, 18% rectal; Cancer Stage 2, 44; Stage 3, 33). All tested tumors had CDH3 expression levels over 0.1% of the PLC level and tumor to normal colon ratios greater than 1. CDH3 expression was noted in 14/22 normal organ tissues. There was a positive correlation between tumor CDH3 QPCR and preoperative CDH3 blood levels (n=57, P= 0.038). Expression levels were significantly higher in rectal vs. colon tumors (p=0.019). Conclusion: CDH3 expression was elevated in CRC tumors as compared to normal tissue. CDH3 was expressed by numerous normal organs and, thus, is not a viable vaccine target, however, the correlation between plasma and tumor CDH3 levels suggests CDH3 may have value as a diagnostic or prognostic marker.

## INTRODUCTION

Colorectal cancer (CRC) is the 3rd most common cancer in males and females in the United States and is the 2nd leading cause of death[1]. Numerous investigators have attempted to identify CRC markers that are over or under expressed and to determine if they might have diagnostic, prognostic, or therapeutic value. One avenue of study concerns molecules that control cellular adhesion and binding. This group includes the cadherins which are calcium-dependent transmembrane glycoproteins that mediate cell adhesion [2]. Cadherins, in general, are key adhesion molecules involved in organ development and morphogenesis. Promotor methylation is thought to be the prominent form of cadherin-related transcriptional regulation and disruption. The fact that a 5’-flanking region of cadherins has been characterized as a CpG island supports this assertion since genes associated with CpG islands not uncommonly undergo methylation regulation at promoter sites [3].

Through the interaction of β-catenin, cadherins mediate cellular adhesion and tissue morphology [4, 5]. All family-associated cadherins retain a calcium binding site as well as multiple cadherin tandem repeat domains. Differentiation of cadherins is found in tissue locations whose divergence is determined by differences in ectodomain sites that allow for tissue specificity [6]. P-cadherin is so named because of its presence in murine placenta. The highest expression of P-cadherin in humans is found in basal or lower layers of stratified epithelia; this finding supports its proposed role in cell proliferation and has prompted investigations regarding a possible role in cancer invasion [7].

The gene encoding P-cadherin (CDH3) is located in a six-cadherin cluster in a region on the long arm of human chromosome 16 [8]. Up-regulation of CDH3 expression has been reported in esophageal [9], pancreatic [10], bladder [11], prostate [12], melanoma [13], and breast cancer. [14] In fact, in breast cancer P-cadherin levels were strongly correlated with the aggressiveness of the tumor; this finding suggests that CDH3 may have a role as a prognostic marker [3]. Of note, CDH3 expression levels in colorectal cancer (CRC) remain less well characterized. One study has found ectopic expression of P-cadherin in early stages of CRC, but expression levels have yet to be correlated to cancer stage [15].

Because P-cadherin is highly expressed in numerous malignancies it may have therapeutic potential as a vaccine target provided it is not widely expressed in normal tissues. If uniquely expressed by CRC's, the administration of CDH3 peptides might result in the development of cytotoxic lymphocytes that would target cancer cells expressing CDH3 in an HLA-A2– restricted manner . To date, P-cadherin's expression profile in normal tissues has not been well studied to date.

The main purpose of this study was to conduct an expression survey of CDH3 in paired CRC tumor and normal colon mucosa samples and to determine the relationship between P-cadherin expression levels and cancer stage as well as preoperative plasma CDH3 levels. A secondary aim was to assess CDH3 expression in a variety of non-colonic normal human tissues in order to evaluate CDH3's potential value as a vaccine target.

## MATERIALS AND METHODS

### Study population and specimen sampling and processing

The CRC plasma and tumor specimens used for this study were obtained from an IRB approved tissue and data bank at New York Presbyterian Hospital, Columbia University Heath Science Campus and Mount Sinai West Hospital in New York City from consenting patient's with colorectal cancer who underwent elective tumor resection. Enrolled patients underwent surgery alone and did not receive a novel drug or other therapy perioperatively. Immunosuppressed and patients who had perioperative blood transfusion were excluded. Likewise, patients who underwent emergent operations were also excluded. Clinical, standard pathologic, and other data was obtained from the prospective data base mentioned above as well as from hospital, operative, and office charts. Preoperative blood specimens were obtained from all patients in the operating room prior to the start of the surgery. Blood samples were stored in a 4°C refrigerator and processed within 2 hours. The plasma aliquots were stored in a −80°C freezer before further analyses. Samples of tumor and grossly normal mucosa (taken at least 2cm away from the tumor) were obtained from the colorectal specimens after the bowel was opened and examined by a pathologist shortly after resection. Tissue samples were embedded in OCT compound (Tissue Tek, Torrance, CA), and stored in a −80°C freezer.

### Initial tissue evaluation

Candidate frozen tumor and normal colonic sections were evaluated histologically to confirm the presence of tumor before being included into the study. The analysis was done at the Irvin Cancer Center of Colombia University in New York City. Poor quality specimens were excluded from the study.

### RNA extraction

Frozen tissue specimens in OCT blocks were sectioned into 10 μm thick sections which were placed into tubes and homogenized by TissueLyser II (Qiagen, CA) with 1 ml Qiazol (Qiagen, CA) added. After the aqueous phase was recovered from the chloroform-derived phase separation, another step of acid phenol-chloroform extraction was performed with a phase lock gel tube (Qiagen, CA). RNA precipitation, column binding, on- column DNase digestion, washing, and elution were performed according to manufacturer's instructions. RNA integrity was confirmed by agarose gel electrophoresis and the concentration determined by measuring the absorption at OD260nm in a BioPhotometer (Eppendorf, NY).

### Reverse transcription

The cDNA first strand reverse transcription was performed with the ABI High Capacity RNA-to-cDNA kit (Applied Biosystems, CA). Briefly, 1 μg of total RNA, 10 μl of 2× reverse transcription buffer, and 1 μl of 20× enzyme mix were incubated together in a total volume of 20 μl at 37°C for 60 minutes, followed by 95°C for 5 minutes, and held at 4°C. The synthesized cDNA was stored at –20°C until further use.

### Quantitative PCR

Comparative quantitative PCR was performed in an Mx3005P real-time PCR machine (Stratagene, CA), using the QuantiTect SYBR Green PCR kit (Qiagen, CA). Briefly,PCR was carried out in a 20 μl volume in a final concentration of 1× reaction buffer containing 300 nM forward and reverse primers, and 10 ng cDNA. Information about forward and reverse primers for P-Cadherin (CDH3) are provided in Table 1. The PCR reaction steps were as follows: hot-start at 95°C for 15 minutes, and then 45 cycles of 95°C for 20 seconds, followed by 55°C for 30 seconds and 72°C for 30 seconds, after which a dissociation curve measurement from 55°C to 95°C was carried out. All samples were done at least in duplicate. PCR data was analyzed using software MxPro (Stratagene, CA). Comparative quantitative analysis was performed based on a delta-delta Ct method using actin gene as an internal control. Results were expressed as relative quantity (dRn). dRn is the magnitude of the fluorescence signal generated during the PCR at each time point that normalized to the reference dye after subtraction of background. Each plate contains amplification on testis and placenta cDNA template which is an intra-/cross-plate calibrator.

**Table 1:** P-Cadherin (CDH3) primer information

Primer ID	Primer name	Sequence	Start	Tm	Location	Product size	SNP
396	CDH3-AF	tgaccacaagcccaagtttac	1759	60.02	exon6	138 bp	None
397	CDH3-AR	taagcaaccaccccattgtag	1896	59.87	exon7		None

### Semi-quantitative RT-PCR

#### RNA samples

Pooled RNAs from normal tissues (testis, placenta, bladder, brain, breast, colon, small intestine, heart, kidney, leukocytes, liver, lung, skeletal muscle, ovary, pancreas, prostate, spleen, stomach, thymus, thyroid, uterus) were purchased from Clontech laboratories, Inc. (Palo Alto, CA) and Ambion, Inc. (Austin, Texas).

#### Reverse-transcription PCR

RNA samples (1 µg of commercial RNA from normal tissues) were reversed transcribed in a total volume of 20µl using Omniscript RT kit (Qiagen, Valencia, CA) according to the manufacturer's protocol using oligo(dT)12-18 primer and RNaseOUT (Invitrogen, Carlsbad, CA). The cDNA was diluted five times with nuclease free water, and 3 µl of diluted cDNA was used in 25µl PCR reactions. For amplification, JumpStartTMREDTaq RedyMix (Sigma Aldrich, St.Louis, MO) and 10 pmol of each primer were used. Primers used for amplification were compiled from the literature or were designed using Primer3 software (http://frodo.wi.mit.edu/cgi-bin/primer3/primer3_www.cgi). Primers were designed to have an annealing temperature around 60°C and to encompass introns, thus allowing product discrimination in the case of genomic DNA amplification. Primer specificity was confirmed by aligning with the NCBI sequence database using BLAST http://blast.ncbi.nlm.nih.gov/Blast.cgi. The amplification protocol used was as follows: precycling hold at 95°C for three minutes followed by 35 cycles of denaturation at 95°C for 15 seconds, annealing for 30 seconds (10 cycles at 60°C, ten cycles at 58°C and 15 cycles at 56°C) and extension at 72°C for 30 seconds followed by a final extension step at 72°C for 7 minutes. PCR products were separated on 1.5% agarose gels stained with ethidium bromide.

### Plasma P-cadherin assays

The plasma levels of P-cadherin were determined via ELISA according to the manufacturer's protocol, in duplicate samples, using a human P-cadherin ELISA duo set kit (R&D Systems, Minneapolis, MN) and a Synergy 2 microplate reader (Biotek, Winooski, CT).

### IHC analysis

Prior to the immunohistochemistry (IHC) studies, 5 mm-thick tissue sections obtained from randomly selected frozen blocks from the study population were stained with Hematoxylin and Eosin (H&E). Presence of cancer and normal tissue on slides was confirmed by a pathologist. To conduct IHC staining, antigen retrieval was performed by heating in a 10 mM citrate buffer (pH 6.0). After epitope recovery, slides were incubated with a 1:250 dilution of Mouse Anti-Human P-Cadherin Monoclonal Antibody (MAB861,R&D Systems, MN) overnight at 4°C. Slides were washed and incubated with secondary biotinylated goat-anti- IgG at 1:500 dilution and tertiary streptavidin- peroxidase conjugate (ABC complex, Vector Laboratories, Inc.). Slides were then treated with chromogen “diaminobenzidine” for antigen detection. Counterstaining was performed with Hematoxylin, dehydrated, cleared in xylene, and mounted on coated slides.

### Statistical analysis

The age of study subjects in the demographic table is expressed as the mean and standard deviation, whereas gender and tumor stages were shown as percentages. To gain insight into the relationship between P-cadherin qPCR expression levels and P-cadherin plasma levels, a nonparametric Spearman's Rho test, which tests for correlation between two rank-ordered variables, was used. A p value <0.05 was considered statistically significant. Expression levels in colon and rectal tumor groups were compared using Mann-Whitney test. The relationship between P-cadherin expression levels of different subgroups (nodal and overall cancer stage) was also determined by Mann-Whitney test.

## RESULTS

### Demographics and clinical data

A total of 77 patients with Stage 2 category or 3 category colorectal cancer (colon, 63 [82%]; rectal, 14 [18%]) that underwent surgical resection were included in this study (mean age 67.1+/-14.5 years; males, 36 [47%]; females, 41 [53%]). The stage breakdown was: stage 2, 44 patients (57%); stage 3, 33 patients (63%).

### Expression levels of P-cadherin in normal tissue

**Figure 1:**
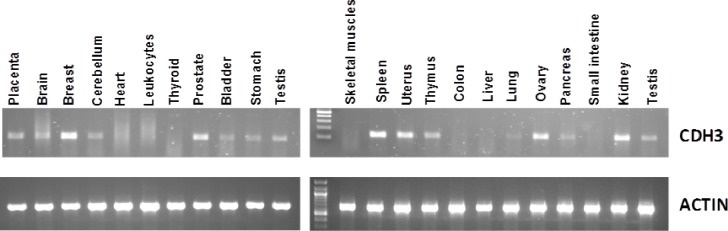
Semi-quantitative analysis of (CDH3) expression in pooled RNA from normal human tissues

The expression of CDH3 was evaluated in 22 normal adult tissues using semi-quantitative RT-PCR. Expression of this gene was found in breast, prostate, spleen, uterus, ovary, kidney, cerebellum, bladder, stomach, testis, thymus, brain and pancreas in addition to the placenta. Expression of P-cadherin was not detected in heart, leukocytes, skeletal muscles, liver, lung, thyroid, small intestine and colon (Figure-1).

### Expression of P-cadherin in colorectal tumor specimens

**Figure 2:**
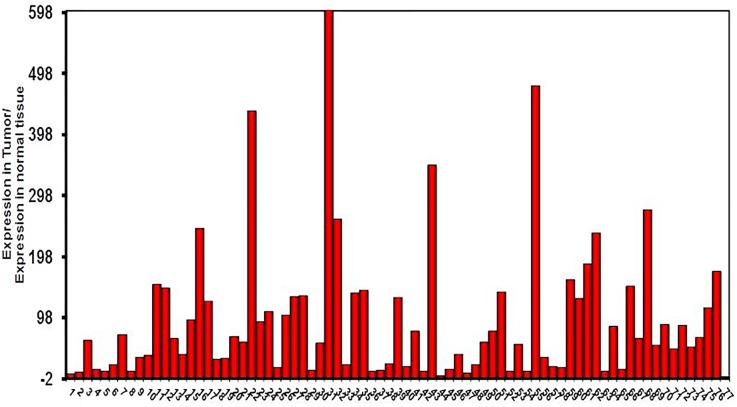
CDH3 relative expression tumor vs. adjacent normal mucosa

All 77 tumors (100%) had tumor/normal colorectal tissue P-cadherin mRNA expression ratios greater than 1 (Figure-2). Also, P-cadherin expression levels in all tumors were greater than 0.1% of placental expression level. Expression ratios in 25 tumors were above 100, ratios in 19 tumors were in the 50-100 range and ratios in 33 tumors were in the 1-50 range (vs their paired normal colon tissues). The P-cadherin IHC analysis of both the tumor and normal tissue specimens was conducted in a subset of patients (n= 18) and confirmed the presence of P-cadherin in CRC tumor tissue and its absence in adjacent normal tissue (Figure-3).

**Figure 3A:**
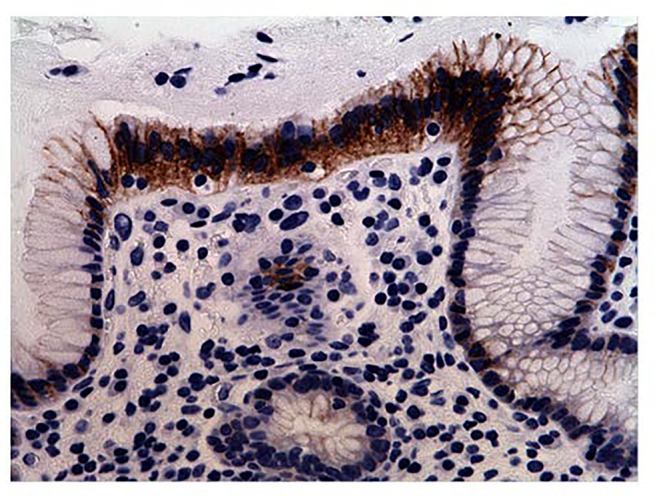
Immunohistochemistry (IHC) staining of gastric-antral mucosa (positive control)

**Figure 3B:**
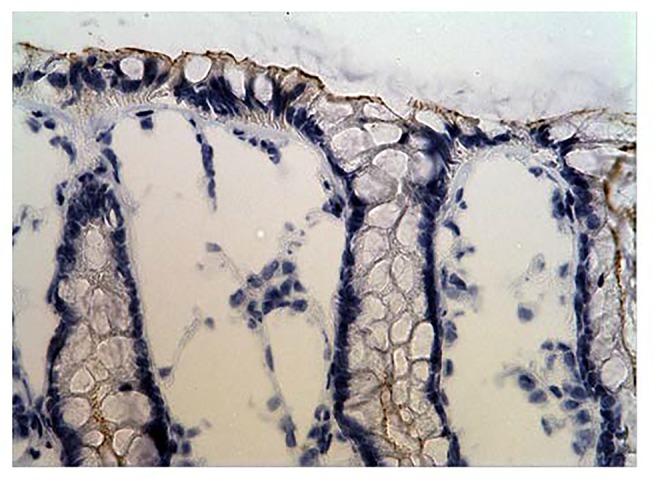
IHC staining of Normal colonic mucosa (negative control)

**Figure 3C:**
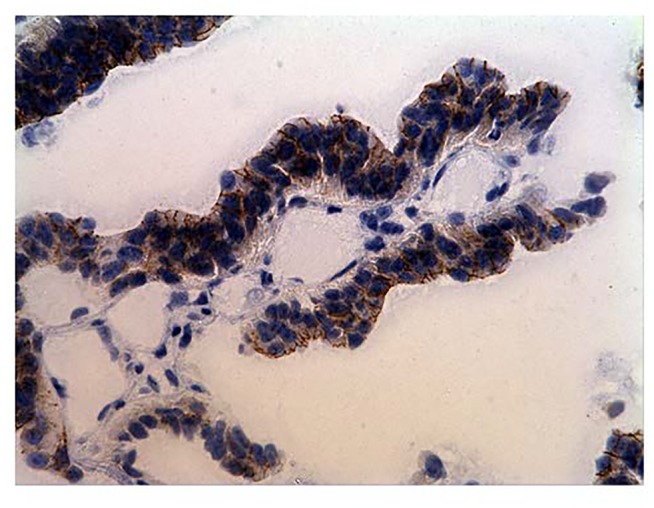
IHC staining of colonic adenocarcinoma

### Serum levels of P-cadherin in patient plasma collected before surgery

**Figure 4:**
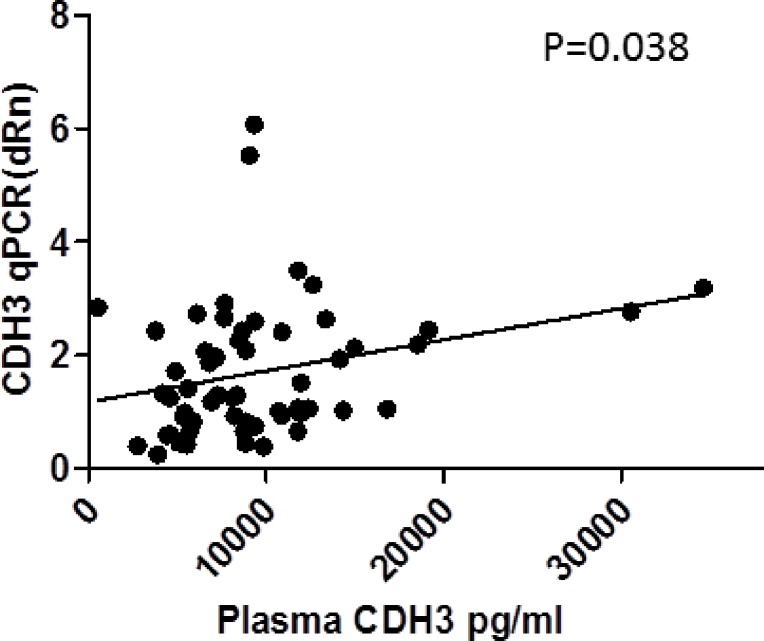
Correlation of CDH3 qPCR (dRn) vs plasma CDH3 levels

In a subset of patients (57/77) plasma P-cadherin levels were determined via ELISA. Relatively higher P-cadherin plasma levels were found in the patients whose tumor specimens showed stronger expression of P-cadherin. There was a significant positive correlation between P-cadherin QPCR and PreOp P-cadherin plasma levels (p= 0.038, correlation coefficient: 0.274, Figure 4).

### P-cadherin gene expression by tumor location & stage

**Figure 5:**
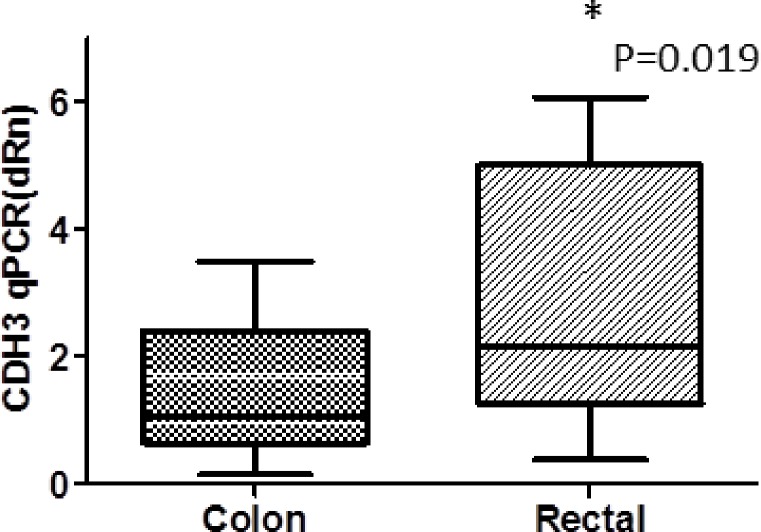
CDH3 qPCR(dRn) colon tumors vs rectal tumors

P-cadherin expression levels were significantly higher in rectal vs. colon tumors (2.17 CI 1.63-6.81 vs 1.06 CI 1.23-1.71; p=0.019, Figure 5). Non-significant increases in P-cadherin mRNA expression levels were noted in Stage 3 vs. Stage 2 tumors and, in the node positive group vs. node negative group (data not shown).

## DISCUSSION

In a variety of human malignancies, tumor progression has been observed to be associated with changes in cell adhesion molecule expression including the cadherin superfamily. Decreased expression levels or loss of E-cadherin function has been implicated in cancer progression and metastasis. E-cadherin down regulation decreases the strength of cellular adhesion within a tissue, resulting in an increase in cellular motility. This in turn may allow cancer cells to cross the basement membrane and invade surrounding tissues [16]. P-cadherin, although structurally similar to classical cadherins, is different from E-cadherin and N-cadherin in terms of immunological specificity and functions[17]. The role of P-cadherin in tumorigenesis is probably tumor type dependent. For example, the progressive loss of normal E-cadherin and P-cadherin expression from melanocytes allows cells to invade and migrate. This releases them from the control of keratinocytes and enables interaction with fibroblasts and vascular endothelial cells in the case of melanoma [18, 19]. However, in infiltrating ductal breast carcinomas, P-cadherin expression correlated significantly with a reduction in E-cadherin expression. Because of this, P-cadherin is believed to enhance cell invasion and tumor aggressiveness, particularly in breast cancer [3].

In our current study, while expression levels were not detectable in archived human colon specimens and normal operative colorectal mucosa, high expression levels were found in colorectal cancer samples. Production of P-cadherin protein was also confirmed by strong IHC staining in a subset of the colorectal tumor samples that were assessed. Similarly, as regards P-cadherin mRNA expression, profoundly increased levels were noted in a subset of patients. It is reported that in colon cancer, P-cadherin mediates defective cell-cell adhesion and enhances anchorage-independent growth, which promotes remote tumor cell seeding [11].

Furthermore, the process of crypt fission has been shown to be a dominant mechanism in the formation of adenomas [20]. A study that utilized murine models found that P-cadherin expression was associated with increased intestinal crypt fission [15]. Thus, the upregulation of P-cadherin may not only be associated with invasive colorectal cancers, but it may also play a role early in the formation of adenomas. Our study shows that P-cadherin expression is elevated in colorectal cancer tissue in contrast to normal colorectal tissue. These results raise the possibility that P-cadherin may play a role in colorectal tumorigenesis.

As mentioned in the introduction, tumor-associated antigens that are highly expressed in tumors but not in normal tissues are tumor vaccine candidates. If a tumor associated antigen is expressed in normal tissues it is highly unlikely that it will elicit a meaningful immune response. Although P-cadherin has been shown to be expressed in a wide variety of different tumors, to date, expression levels in normal human tissues had not been well studied. In the current study we assessed P-cadherin expression in 22 different human tissues and found that it was expressed in 14 (including placenta) but not in the colon or small bowel. Because it is expressed widely in normal tissues, unfortunately, P-cadherin is not a vaccine candidate.

Although P-cadherin does not appear to be a viable vaccine target, it may hold potential as a prognostic marker. In our study, we show a positive correlation between preoperative P-cadherin plasma levels and P-cadherin expression in colorectal tumor tissue (Figure 4). In terms of staging, levels of P-cadherin were slightly higher in patients with stage III disease with lymph node metastasis compared to those with stage II disease without lymph node metastasis, although the increase was not statistically significant. Further studies with larger patient numbers stratified according to cancer stage are necessary to determine whether P-cadherin expression correlates with tumor stage, lymph node metastases or other pathologic parameters. Similarly, larger studies that assess P-cadherin blood levels both preoperatively and postoperatively at the time of follow up visits would make it possible to determine if monitoring of serum P-cadherin levels holds promise as a means of diagnosing tumor recurrences. It is also possible that P-cadherin could be paired with other blood tumor markers for the purposes of diagnosing recurrences or, perhaps, the primary tumor. In this study it was also shown that P-cadherin expression was significantly higher in rectal tumors vs colon cancers raising the question as to whether it may play a greater role in the development of rectal cancer. Further study is needed to verify these results and to determine their significance.

### Summary

All 77 tumors assessed had P-cadherin tumor/normal tissue expression ratios that were greater than 1 and also greater than 0.1% of placental levels, thus colorectal cancers can be said to express P-cadherin. IHC analysis on a subset of patients confirmed the presence of protein in the tumor sections. Further, plasma P-cadeherin levels were found to directly correlate with tumor expression levels and levels were higher in stage 3 vs stage 2 tumors (p=ns). P-cadherin expression was found in 14/22 normal organ tissues tested. Thus, P-cadherin is not a promising vaccine target. However, P-cadherin may still prove useful as a prognostic CRC tumor marker, particularly for rectal cancer. A larger and more diverse group of tumors (Stage I - IV) needs to be assessed to determine if P-cadherin expression correlates with tumor size, lymph node metastasis, or final tumor stage. Assessment of blood P-cadherin levels in a larger population of CRC patients as well as in a benign patient population is also warranted.
